# Mites of the family Bdellidae Dugès (Trombidiformes, Bdelloidea) from Saudi Arabia: a new species, new records, and a key to the world species of the genus *Cyta* von Heyden

**DOI:** 10.3897/zookeys.1284.199197

**Published:** 2026-07-14

**Authors:** Jawwad Hassan Mirza, Fahad Jaber Alatawi

**Affiliations:** 1 Department of Plant Protection, College of Food and Agriculture Sciences, King Saud University, Riyadh, Saudi Arabia Department of Plant Protection, College of Food and Agriculture Sciences, King Saud University Riyadh Saudi Arabia

**Keywords:** *
Bdella
*, *
Cyta
*, *
Hexabdella
*, *
Odontoscirus
*, species groups, taxonomy

## Abstract

Four bdellid genera, *Bdella* Latreille, *Hexabdella* van Der Schyff, Theron & Ueckermann, *Odontoscirus* Thor, and *Tetrabdella* Hernandes & Feres, are reported for the first time from Saudi Arabia, as are eight species, *B. tropica* Atyeo, *H. maraugia* van Der Schyff, Theron & Ueckermann, *O. longirostris* (Hermann), *Spinibdella subrufa* Rack, *S. tabarii* Paktinat-Saeej & Bagheri, *S. tadjikistanica* Kuznetsov, *S. thori* (Meyer & Ryke), and *T. neotropica* Hernandes & Feres. Additionally, *Cyta edafosa***sp. nov**. is described and illustrated based on the female. Furthermore, through a comprehensive analysis of the published literature, species of *Cyta* von Heyden are categorized into three species groups based on the presence or absence of trichobothrium on the leg tibiae I, IV, and tarsus III. Taxonomic notes on some *Cyta* species, along with an identification key to known *Cyta* species, are also provided.

## Introduction

The mites of the family Bdellidae Dugès, 1834 (Trombidiformes, Bdelloidea) are relatively large, soft-bodied predators of small arthropods (Hernandes et al. 2016). The family is comprised of five subfamilies, which are mainly differentiated based on the number of ventral setae on the subcapitulum and the presence or absence of a trichobothrium on leg tibia II and tarsus IV. Bdellid mites inhabit a variety of habitats and are reported from a wide range of climatic regions worldwide (Hernandes et al. 2016). A few species have cosmopolitan distribution, i.e. *Cyta latirostris* (Hermann, 1804) and *Neomolgus littoralis* (Linnaeus, 1745), and some species are of predatory significance, including *Bdella tropica* Atyeo, 1960 and *Spinibdella cronini* (Baker & Balock, 1944) (Hernandes et al. 2016). Hernandes et al. (2016) produced a comprehensive systematic work that included keys to subfamilies, genera, and species of the Bdellidae.

Species of *Cyta* von Heyden are characterized by short, stout chelicerae with inflated bases, the presence of two setae on the ventral hypostome, and the absence of a trichobothrium on tarsus IV (Hernandes et al. 2011, 2016; Wu et al. 2021). The genus comprises 22 valid species (Hernandes et al. 2016; Paktinat-Saeij and Kazemi 2025; Mirza and Alatawi 2026), reported from all around the world, except Antarctica (Hernandes et al. 2016). Wallace and Mahon (1972) suggested the division of *Cyta* into three groups based on the presence or absence of trichobothrium on the tibiae I and IV and on tarsus III. However, this proposal for species groups has not been followed in recent taxonomic work (Hernandes et al. 2016; Paktinat-Saeij and Kazemi 2025).

Three species of Bdellidae are known from Saudi Arabia: *Spinibdella cronini*, *S. bifurcata* Atyeo, 1960, and *Cyta arabica* Mirza & Alatawi (Soliman and Al-Yousif 1979; Al-Khalifa and Bayoumi 1983; Alatawi 2011; Alatawi and Kamran 2017; Mirza and Alatawi 2026). The present study explores the diversity of the bdellid fauna in Saudi Arabia and provides a comprehensive literature-based analysis of *Cyta* species.

## Materials and methods

Specimens of mites were collected by: (1) shaking the plant foliage over a white sheet of paper and preserving it in small vials with 70% ethanol, or (2) collecting the soil debris and processing it through a Berlese funnel. Specimens were mounted on a glass slide in Hoyer’s medium following Walter and Krantz (2009) and identified under a phase-contrast microscope (BX51, Olympus, Tokyo, Japan). Photographs of body parts for morphological characterization were captured using the Automontage software system (Syncroscopy, Cambridge, UK) attached to a phase-contrast microscope (DM2500, Leica, Wetzlar, Germany). Additionally, the subcapitulum, chelicerae, and all legs were hand-drawn using a camera lucida attached to the phase-contrast microscope. The photographs and drawings were traced in Adobe Illustrator (Adobe Systems Inc., San Jose, CA, USA) to produce the final figures.

Body length was measured from the base of gnathosoma to the posterior margin of the idiosoma, and body width at the level of setae *c_2_*; setae were measured from their base to the tip. Legs were measured from the base of the trochanter to the tip of the pretarsal claw. All measurements are given in micrometres (μm) and variations of leg setal number in parentheses. The holotype and three paratypes have been deposited in the King Saud University Museum of Arthropods (**KSMA**, Acarology section), Department of Plant Protection, College of Food and Agriculture Sciences, King Saud University, Riyadh, Saudi Arabia. The two paratypes with Accession numbers, OSAL 162750 and OSAL 162751 were deposited at Ohio State University Acarology Laboratory (OSAL), USA

The dorsal and ventral setal nomenclature follows Paktinat-Saeej et al. (2015) and Fisher et al. (2011), and the leg setal notation follows Den Heyer (1981). The abbreviations of setal names are as follows: anterior trichobothria (*at*), posterior trichobothria (*pt*), lateral proterosomal setae (*lps*), median proterosomal setae (*mps*), internal humerals (*c_1_*), external humerals (*c_2_*), internal dorsals (*d_1_*), internal lumbals (*e_1_*), internal sacrals (*f_1_*), external sacrals (*f_2_*), internal clunals (*h_1_*), external clunals (*h_2_*), postanals (*ps*), anal setae (*ad*), aggenital setae (*ag*), genital setae (*g*), ventral hypostomal setae (*vh_1_*_–2_), dorsal hypostomal setae (*DHS*), adoral setae (*or_1_*_–2_), attenuate (sharply) solenidion (*asl*), blunt-pointed rod-like solenidion (*bsl*), trichobothrium (*T*), simple tactile seta (*sts*), unpaired median seta (*ums*), solenidion (*s*), dorsal end seta (*DES*), and ventral end seta (*VES*).

## Results

Four genera of Bdellidae are reported for the first time from Saudi Arabia. These represent three subfamilies: *Tetrabdella* Hernandes & Feres, 2006 (Spinibdellinae); *Odontoscirus* Thor, 1913 (Odontoscirinae); and *Bdella* Latreille, 1795 and *Hexabdella* van Der Schyff, Theron & Ueckermann, 2004 (Bdellinae). Eight species are newly reported as part of the bdellid mite fauna of Saudi Arabia: *B. tropica*; *H. maraugia* van Der Schyff, Theron & Ueckermann, 2004; *O. longirostris* (Hermann, 1804); *Spinibdella subrufa* Rack, 1961; *S. tabarii* Paktinat-Saeej & Bagheri, 2015; *S. tadjikistanica* Kuznetsov, 1984; *S. thori* (Meyer & Ryke, 1959); and *T. neotropica* Hernandes & Feres, 2006. Additionally, *C. edafosa* sp. nov. is described and illustrated based on the female.

*Cyta* species have been previously suggested to be categorized into three groups based on the presence or absence of the trichobothria on three leg segments: (1) leg tibiae I, IV, and tarsus III each with a trichobothrium; (2) only tibia IV with a trichobothrium; and (3) trichobothrium absent on all legs (Wallace and Mahon 1972). However, it is assumed that due to the absence of an adequate number of representative species, this grouping was not followed (Hernandes et al. 2016). In the recent work of Paktinat-Saeij and Kazemi (2025), three species (*C. latirostris*, *C. kurdistanicus* Eghbalian, Khanjani & Ueckermann (in Eghbalian et al. 2014), and *C. kreiteri* Barbar & Ueckermann, 2017) were classified as belonging to group 2, and a species described by these authors (*C. akhyanii* Paktinat-Saeij & Kazemi, 2025) was only included in group 3. In Bdellidae, the presence or absence of leg trichobothria has taxonomic significance at subfamilial and generic levels (Hernandes et al. 2016).

In the present study, a critical analysis of published literature of all known *Cyta* species (*n* = 22, excluding the new species described here) revealed a significant number of representative species in each group. The three groups, *sensu*Wallace and Mahon (1972), are hereby implied to categorize the *Cyta* species into three species groups. Consequently, 11 species belong to *coerulipes* species group, seven species (including the new species) belong to *latirostris* species group, and three species belong to *veneta* species group. Two species, *C. cytoides* (Mihelčič, 1958), and *Cyta flava* Mihelčič, 1958, were poorly described and inadequately illustrated; hence, neither species was placed in a species group nor included in the diagnostic key. Notes on both these species, in addition to the status of *C. akhyanii*, *C. grandjeani* Gomelauri, 1963, and *C. veneta* (Lombardini, 1960), are provided. An updated key to the *Cyta* species of the world is also developed.

### New species

**Family Bdellidae Dugès, 1834**

**Subfamily Cytinae Grandjean, 1938**

#### Genus Cyta

Taxon classificationAnimaliaTrombidiformesBdellidae

von Heyden, 1826

7EC18414-8036-5DB8-816A-A63DABE63026

##### Type species.

*Scirus latirostris* Hermann, 1804: 62 by original designation.

#### Cyta edafosa sp. nov.

Taxon classificationAnimaliaTrombidiformesBdellidae

910FEC2C-918F-5BD5-A2D5-D9895D0F5CFA

https://zoobank.org/7ABE474D-83D5-4D42-ABC1-B39CFE29185A

Figs 1, 2, 3, 4, 5, 6, 7, 8, 9

##### Type material.

Saudi Arabia • ***holotype***: ♀; Jazan, AlHasher mountain; 17°27.220'N, 43°3.130'E; 23 Feb. 2025; E.M. Khan, N.A. Elgoni and H.M.S. Ali leg.; soil under *Buddleja polystachya* (Scrophulariaceae); KSMAAS25-Bde-Cyt-H. ***Paratypes***: • 3 ♀; same data as holotype; KSMAAS25-Bde-Cyt-P1-5. • 2 ♀; same data as holotype; OSAL 162750 and OSAL 162751, deposited at Ohio State University Acarology Laboratory (OSAL), USA

##### Diagnosis.

Dorsum entirely covered with broken striations; palp basifemur and leg telofemur each with five setae; trichobothrium present only on leg tibia IV; chelicerae with an edentate movable digit; fixed digit with a tooth; leg trochanter I with two setae; short *mps* setae not reaching base of *lps* setae; coxae and subcapitulum without reticulations.

##### Description.

**Female** (*n* = 6). Body length including gnathosoma 820 (800–830); body length excluding gnathosoma 670 (650–688); width 430 (425–450).

**Figure 1.**
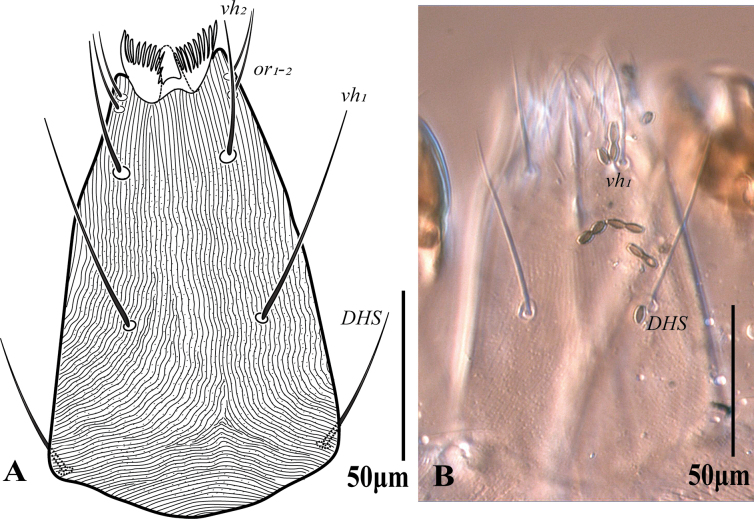
*Cyta edafosa* sp. nov., female. **A**. Subcapitulum; **B**. Microphotograph.

**Figures 2, 3.**
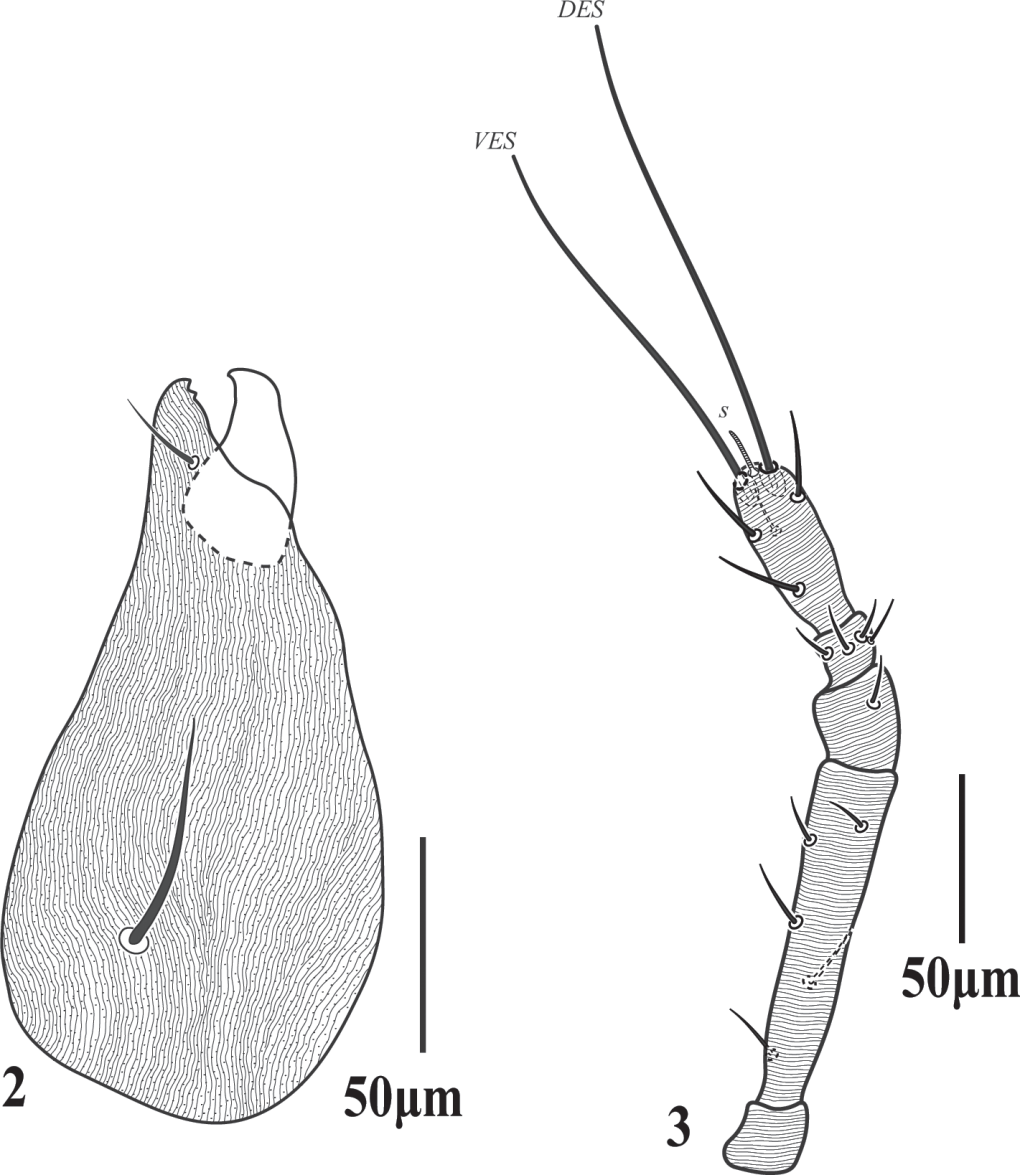
*Cyta edafosa* sp. nov., female. **2**. Chelicera; **3**. Palp.

***Gnathosoma*** (Figs 1, 2, 3). Subcapitulum 160 (155–168) long, width at base 114 (107–120), with pitted, longitudinal striae anterior to setae *vh_1_*, with two pairs of long, ventral setae, *vh_1_* 66 (63–70), *vh_2_* 35 (33–38); distance *vh_1_*_–*2*_ 46 (44–48); two pairs of short adoral setae near tip of subcapitulum, *or_1_* 18 (16–20) and *or_2_* 17 (15–18) (Fig. 1A, B).

***Chelicerae*** (Fig. 2) 165 (160–170) long, 82 (77–86) wide; longitudinal pitted striations from cheliceral base to level of distal seta and chelae with two dorsal setae; proximal seta 66 (62–69); distal seta 24 (22–26); distance between proximal and distal setae 105 (105–118); movable digit edentate and fixed digit with a tooth. Palp trochanter with seta absent; basifemur with five setae; telofemur with one seta; genu with four setae; tibiotarsus with four setae (*sts*), one solenidion (*s*), and two long terminal setae, *DES* and *VES* 144 (140–150) and 115 (111–120), respectively; measurements of palp segments as follows: trochanter 16 (14–18), basifemur 82 (78–85), telofemur 33 (30–35), genu 20 (18–22), tibiotarsus 49 (47–51) (Fig. 3).

**Figure 4.**
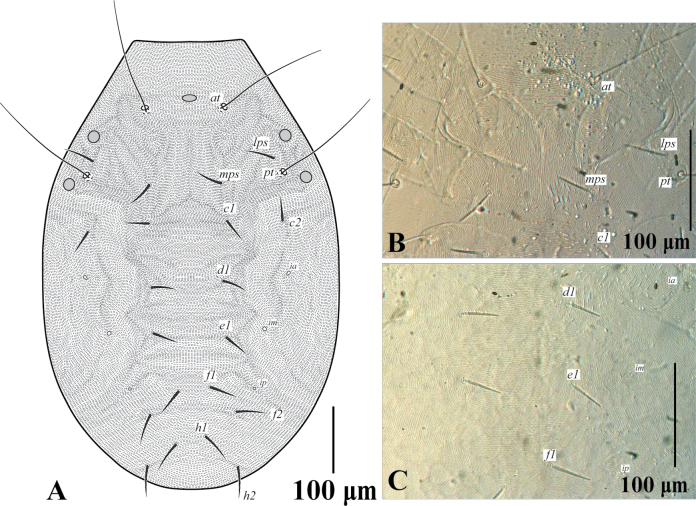
*Cyta edafosa* sp. nov., female. **A**. Dorsum; **B**. Microphotograph of dorsal propodosoma; **C**. Microphotograph of dorsal hysterosoma.

***Dorsum*** (Fig. 4A–C). Dorsal idiosoma entirely by broken striae; prodorsum with transverse striae between setae *at*; longitudinal striae between setae *mps*. Prodorsum with two pairs of lateral eyes and a median unpaired eye; diameters of unpaired median eye 16 (15–17), anterior lateral eye 18 (17–20), and posterior lateral eye 19 (18–21); distance between two lateral eyes 49 (48–52), with longitudinal striae in between. Dorsal setae, other than smooth *at* and *pt*, faintly serrate. Hysterosomal region with three pairs of cupules (*ia*, *im*, and *ip*) at level of setae *d_1_*, *e_1_*, and posterolateral to *f_1_*; striae between setae *c_1_*, *d_1_*, *e_1_*, *f_1_*, *f_2_*, *h_1_*, and *h_2_* transverse, broken, oblique, and irregular laterally. Measurements of dorsal setae as follows: *at* 164 (160–169), *lps* 49 (47–52), *mps* 41 (40–43), *pt* 172 (165–180), *c_1_* 38 (36–42), *c_2_* 36 (34–38), *d_1_* 38 (36–40), *e_1_* 41 (40–43), *f_1_* 44 (42–47), *f_2_* 45 (43–48), *h_1_* 46 (45–48), *h_2_* 44 (42–45). Distance between dorsal setae: *at*–*at* 114 (110–120); *lps*–*lps* 254 (244–265); *at*–*lps* 106 (102–110); *pt*–*pt* 260 (255–275); *mps*–*pt* 90 (86–94); *mps*–*mps* 98 (95–1104); *at*–*pt* 123 (118–130); *at*–*mps* 106 (101–112); *c_1_*–*c_1_* 114 (109–120); *c_1_*–*c_2_* 82 (80–89); *d_1_*–*d_1_* 103 (97–109); *e_1_*–*e_1_* 106 (100–112); *f_1_*–*f_1_* 36 (32–40); *f_2_*–*f_2_* 131 (128–136); *h_1_*–*h_1_* 44 (42–47); *c_1_*–*d_1_* 90 (88–95); *d_1_*–*e_1_* 65 (62–69); *e_1_*–*f_1_* 82 (77–90); *f_1_*–*h_1_* 66 (62–70); *h_1_*–*h_2_* 52 (50–55).

**Figure 5.**
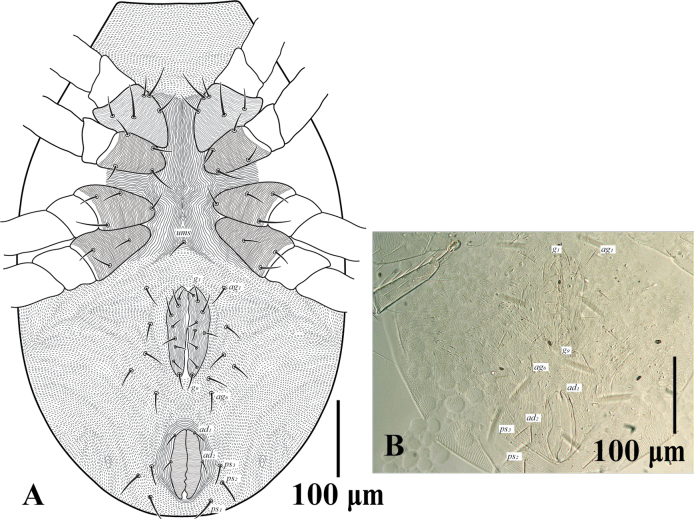
*Cyta edafosa* sp. nov., female. **A**. Venter; **B**. Microphotograph of ventral hysterosoma.

***Venter*** (Fig. 5A, B). Striae broken anterior to coxa I and posterior to coxae IV; area between coxae I–IV with continuous longitudinal striae except central area between coxae II and III with irregular striae; unpaired seta (*ums*) present between coxae IV; aggenital region surrounded by oblique, irregular striae and with six pairs of setae (*ag_1_*_–*6*_) (five pairs on one side in holotype); genital valves each with eight setae (*g_1_*_–*8*_) (nine pairs on one side in holotype); anal region with three pairs of serrated pseudanal setae (*ps_1_*–*_3_*), *ps_1_* 39 (36–42), *ps_2_* 32 (30–34), and *ps_3_* 28 (26–30); anal region at level of seta *ps_2_* with one pair of cupules, two pairs of serrated *ad_1_*_–*2*_ setae.

**Figures 6–9.**
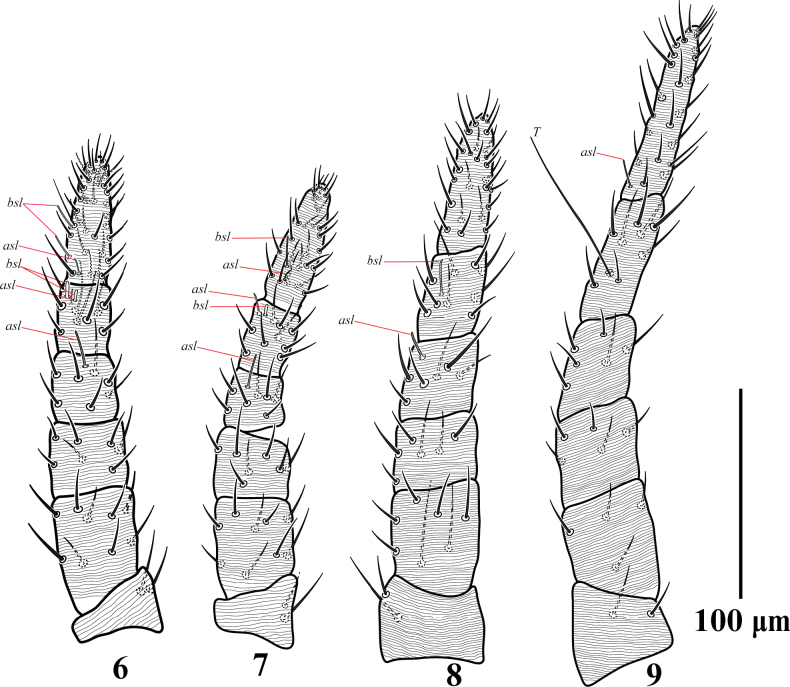
*Cyta edafosa* sp. nov., female. **6**. Leg I; **7**. Leg II; **8**. Leg III; **9**. Leg IV.

***Legs*** (Figs 6–9). Measurements of legs as follows: leg I 314 (302–328), leg II 294 (281–304), leg III 330 (320–340), leg IV 375 (363–385). Setal formulae of leg segments as follows: coxae I–IV 5–4–6(5 setae on right coxa III of holotype; 4 setae in two paratypes)–3 *sts*; trochanters I–IV 2–2–2–2 *sts*; basifemora I–IV 9(8 in one paratype)–9–7–4 *sts*; telofemora I–IV 7(6 in one paratype)–7(6 in one paratype)–6 (7 in one paratype)–5 *sts*; genua I–IV 7*sts*,1*asl*–7*sts*,1*asl*–6*sts*,1*asl*–6*sts* (7*sts* in all paratypes); tibiae I–IV 9*sts*,1*asl*,2*bsl*–9*sts*,1*asl*,1*bsl*–9*sts*,1*bsl*–8*sts*,1*T*; tarsi I–IV 33*sts*,1*asl*,2*bsl*–26*sts*,1*asl*,1*bsl*–25*sts*–23*sts*,1*asl*; only tibia IV with a trichobothrium.

##### Etymology.

This new species was collected from soil, and the specific epithet refers to this (*edafos*, Greek, meaning “soil”).

##### Remarks.

*Cyta edafosa* sp. nov. is morphologically closest to *Cyta grandjeani* (China, Wu & Guo in Wu et al. 2021) due to the presence of only one trichobothrium on leg tibia IV, an edentate movable digit, fixed digit with a tooth, leg trochanter I with two setae, short *mps* setae not reaching the base of the *lps* setae, and coxae and subcapitulum without reticulation. The new species, however, can be distinguished from *C. pseudokreteri* based on the following differences: dorsal integument entirely covered with broken striae vs broken striae present only between dorsocentral setae *c_1_*–*f_1_*, palp basifemur with 5 setae vs 6 setae, leg telofemur IV with 5 setae vs 4 setae, legs comparatively shorter, ranging from 281–385 vs legs very long, ranging from 553–790.

### New records

**Subfamily Bdellinae Dugès, 1834**

#### Genus Bdella

Taxon classificationAnimaliaTrombidiformesBdellidae

Latreille, 1795

E984DACB-C2FF-5EA2-B526-D0BD72835F37

Bdella Latreille, 1795: 18; 1810: 133. Type species: Acarus longicornis Linnaeus, 1758.

#### Bdella tropica

Taxon classificationAnimaliaTrombidiformesBdellidae

Atyeo, 1960

0BB40DC6-21D5-57BD-9F71-514B21114E0C

Bdella tropica Atyeo, 1960: 378.

##### Material examined.

Saudi Arabia • 1 ♀; Jazan, AlHasher mountain; 17°27.220'N, 43°3.130'E; 23 Feb. 2025; E.M. Khan, N.A. Elgoni and H.M.S. Ali leg.; soil under *Buddleja polystachya* (Scrophulariaceae).

##### Distribution.

Malaysia (Shiba 1978), China (Lin et al. 2006), Saudi Arabia (new record).

#### Genus Hexabdella

Taxon classificationAnimaliaTrombidiformesBdellidae

van Der Schyff, Theron & Ueckermann, 2004

1B314F23-293B-58B1-BA5A-4F7E246920FC

Hexabdella van Der Schyff et al., 2004: 14. Type species: Hexabdella denheyeri van Der Schyff, Theron & Ueckermann, 2004.

#### Hexabdella maraugia

Taxon classificationAnimaliaTrombidiformesBdellidae

van Der Schyff, Theron & Ueckermann, 2004

62E31D5B-038F-5652-9959-9797864B38AC

Hexabdella maraugia van Der Schyff et al., 2004: 17.

##### Material examined.

Saudi Arabia • 1 ♀; Asir, Wadi Rahab; 18°9.640'N, 42°15.110'E; 1 Mar. 2024; E.M. Khan leg.; in soil under *Prunus* sp. (Rosaceae).

##### Distribution.

South Africa (van Der Schyff et al. 2004), Saudi Arabia (new record).

###### Subfamily Odontoscirinae Grandjean, 1938

#### Genus Odontoscirus

Taxon classificationAnimaliaEchinostelialesBdellidae

Thor, 1913

E28D5513-E086-57A0-AB14-F5037E7E3F5D

Odontoscirus Thor, 1913: 29.

##### Type species.

*Scirus longirostris* Hermann, 1804 by original designation.

#### Odontoscirus longirostris

Taxon classificationAnimaliaEchinostelialesBdellidae

(Hermann, 1804)

6A92FCC7-7C1D-570B-A78A-521E815882C3

*Sciruslongirostris* Hermann, 1804: 62.
Scirus longirostris —Thor 1904: 75.

##### Material examined.

Saudi Arabia • 1 ♀; Riyadh, Diriyah Plant Nursery; 24°44.790'N, 46°34.709'E, 18 Oct. 2019, J.H. Mirza leg.; on leaf of *Solanum melongena* (Solanaceae).

##### Distribution.

Widely distributed (Hernandes et al. 2016), Saudi Arabia (new record).

###### Subfamily Spinibdellinae Grandjean, 1938

#### Genus Spinibdella

Taxon classificationAnimaliaTrombidiformesBdellidae

Thor, 1930

067513DD-6DE0-55EA-BDC1-E7D3D394057E

Spinibdella Thor, 1930: 22. Type species: Spinibdella reducta Thor, 1930, by original designation.

#### Spinibdella tabarii

Taxon classificationAnimaliaTrombidiformesBdellidae

Paktinat-Saeej & Bagheri, 2015

D4BFAC80-2CD9-53EC-B1C0-14B3B6F4F121

Spinibdella tabarii Paktinat-Saeej & Bagheri in Paktinat-Saeej et al. 2015: 696.

##### Material examined.

Saudi Arabia • 5 ♀; Jazan, Alarida; 17°1.740'N, 43°8.320'E, 22 Feb. 2025; E.M. Khan, N.A. Elgoni and H.M.S. Ali leg.; in soil under *Ficus* sp. (Moraceae).

##### Distribution.

Iran (Paktinat-Saeej et al. 2015), Saudi Arabia (new record).

#### Spinibdella tadjikistanica

Taxon classificationAnimaliaTrombidiformesBdellidae

Kuznetsov, 1984

EA98C198-90FF-5FFF-83FA-998729C1584C

Spinibdella tadjikistanica Kuznetsov, 1984: 774.

##### Material examined.

Saudi Arabia • 1 ♂; Riyadh; 24°48.000'N, 46°42.000'E; 11 May 2011; M. Kamran, J.H. Mirza and H.M.S. Mushtaq leg.; on grasses (Poaceae) • 1 ♂; Tabuk, Tayma; 27°38.008'N, 38°33.850'E; 1 Oct. 2020; J.H. Mirza, H.M.S. Mushtaq and E.M. Khan leg.; *Arundo donax* (Poaceae) • 1 ♂, Jouf, Watania; 29°55.830'N, 38°33.932'E; 21 Jun. 2021; J.H. Mirza leg.; on grasses (Poaceae) • 2 ♀; Tabuk; 28°24.340'N, 36°40.654'E; 23 Jun. 2021; J.H. Mirza leg.; on *Phoenix dactylifera* (Arecaceae) • 1 ♀, 1 ♂; Madina, Al Ula; 26°40.53'N, 38°03.12'E; 20 Mar. 2022; M. Kamran and N.A. Elgoni leg.; on grasses (Poaceae) • 1 ♀; Tabuk, Deesa; 27°35.49'N, 36°25.03'E; 23 Mar. 2022; M. Kamran and N.A. Elgoni leg; on *Haloxylon* sp. (Amaranthaceae) • 2 ♀, 2 ♂; Tabuk, Deesa,; 27°38.11'N, 36°28.52'E; 23 Mar. 2022; M. Kamran and N.A. Elgoni leg; on grasses (Poaceae) • 1 ♀; Jazan, Wadi Alrough; 17°1.750'N, 43°8.310'E; 23 Feb. 2024; N.A. Elgoni leg.; on *Lawsonia* sp. (Lythraceae) • 1 ♂; Madina, Smad; 25°30.418'N, 39°21.389'E; 25 Aug. 2020; F.J. Alatawi, J.H. Mirza and E.M. Khan leg.; on grasses under *Citrus* sp. (Rutaceae) • 1 ♂; Madina; 25°30.418'N, 39°21.389'E; 25 Aug. 2020; F.J. Alatawi, J.H. Mirza and E.M. Khan; on *Cactus* (Cactaceae) • 1 ♂; Madina; 24°35.897'N, 39°23.875'E; 25 Aug. 2020; F.J. Alatawi, J.H. Mirza and E.M. Khan leg.; on *Phoenix dactylifera* (Arecaceae).

##### Distribution.

Iran (Rostami et al. 2017), Tadjikistan (Kuznetsov 1984), Saudi Arabia (new records).

#### Spinibdella thori

Taxon classificationAnimaliaTrombidiformesBdellidae

(Meyer & Ryke, 1963)

282663CF-48BE-5BEC-9E5C-6334C4ECC7E8

Bdella thori Meyer & Ryke, 1959: 375.
Spinibdella thori —Atyeo 1963: 174.

##### Material examined.

Saudi Arabia • 3 ♀; Jazan, Fayfa; 17°15.120'N, 43°6.352'E, 23 Feb. 2025; E.M. Khan, N.A. Elgoni and H.M.S. Ali leg.; in soil under *Ziziphus* sp. (Rhamnaceae).

##### Distribution.

South Africa, Australia, Hawaii, Mexico, Iran (Hernandes et al. 2016), Saudi Arabia (new record).

#### Spinibdella subrufa

Taxon classificationAnimaliaTrombidiformesBdellidae

Rack, 1961

07126097-4FBE-5AB7-9188-F24FA618D0A2

Spinibdella subrufa Rack, 1961: 183.

##### Material examined.

1 ♀; Qassim; 26°23.010'N, 44°13.140'E, 3 May 2018; M. Kamran, J.H. Mirza, and H.M.S. Mushtaq leg; in soil under *P. dactylifera* (Arecaceae) • 1 ♂; Riyadh, Hota Bani Tamim; 23°12.380'N, 46°29.106'E; 8 May 2018; Kamran, J.H. Mirza, and H.M.S. Mushtaq leg; on *P. dactylifera*.

##### Distribution.

Germany (Rack 1961), Saudi Arabia (new record).

#### Genus Tetrabdella

Taxon classificationAnimaliaTrombidiformesBdellidae

Hernandes & Feres, 2006

B9E34DC5-442A-5564-99A3-8DA70CC32B60

Tetrabdella Hernandes & Feres, 2006:60.

##### Type species.

*Tetrabdella neotropica* Hernandes & Feres, 2006 by original designation.

#### Tetrabdella neotropica

Taxon classificationAnimaliaTrombidiformesBdellidae

Hernandes & Feres, 2006

094B631D-8F57-5811-97BC-671C43E3577B

Tetrabdella neotropica Hernandes & Feres, 2006: 60.

##### Material examined.

1 ♀; Jazan, Abu Arish; 16°56.330'N, 42°49.660'E; E.M. Khan, N.A. Elgoni, and H.M.S. Ali; on *Mangifera* sp. (Anacardiaceae) • 1 ♀; Riyadh, Alfalah; 24°48.00'N, 46°42.00'E; 11 May 2011; M. Kamran, J.H. Mirza, and H.M.S. Mushtaq; on *Senna* sp. (Fabaceae) • 2 ♀; Jazan, Alaridah; 17°03.499'N, 42°57.664'E, 8 Oct. 2020; J.H. Mirza, H.M.S. Mushtaq, and E.M. Khan leg; on *Mangifera* sp. (Anacardiaceae) • 1 ♀; Jazan, Alaridah; 17°03.499'N, 42°57.664'E, 8 Oct. 2020; J.H. Mirza, H.M.S. Mushtaq and E.M. Khan leg.; on *Acacia* sp. (Fabaceae) • 3 ♀; Jazan, Wadi Alrough; 17°1.75'N, 43°8.320'E; 23 Feb. 2024; N.A. Elgoni leg.; on *Amaranthus* sp. (Amaranthaceae).

##### Distribution.

Brazil (Hernandes and Feres 2006), Mexico (Gallardo-Yobal et al. 2025), Saudi Arabia (new records).

## Discussion

### Notes on some *Cyta* species

Species group *veneta*

This species group currently includes three species, *C. akhyanii*, *C. grandjeani*, and *C. veneta*. *Cyta veneta* (Lombardini) was originally described as *Neomolgus venetus* Lombardini, 1960 based on specimens collected from coastal dunes at Jeloso, Italy (“dalle dune costiere di Venezia (Jesolo)”; Lombardini 1964). Later, it was transferred to *Cyta* upon re-examination of the specimen (Lombardini 1964). In the same publication, Lombardini (1964) described a new subspecies, *C. veneta grandjeani*, based on specimens collected at Kilyos, Turkey, along the Black Sea coast (“su le dune di Kylios (Costa turca del Mar Nero)”) that were larger (920 μm vs 760 μm) and had narrower chelicerae (length-to-width ratio of 2.33 vs 1.61) compared to the nominate subspecies (Lombardini 1964). *Cyta veneta grandjeani* was later considered a junior homonym of *C. grandjeani*, so the name was suppressed (Hernandes et al. 2016). Lombardini (1964) stated that leg trichobothria were absent in *C. veneta* and is so included here in the *veneta* species group. It can be differentiated from *C. akhyanii* based on leg chaetotaxy of basifemora I–III (6–6–6 vs 8–8(9)–6), solenidion on leg genu II (absent vs present), and teeth on fixed cheliceral digit (absent vs three teeth).

Grandjean (1938) reported two possible species of *C. latirostris* in France, the typical species with a trichobothrium present only on tibia IV, and a second, undescribed species with leg trichobothria are entirely absent. Grandjean reported other differences between these two but did not describe the latter due to a lack of specimens. Atyeo (1960) examined only the specimens of the typical *C. latirostris* and re-described it. Gomelauri (1963) later described *C. grandjeani* from Tiflis (now Tbilisi) and Abkhazia (Georgia) and considered it similar to the undescribed species of Grandjean (1938). Gomelauri’s work was not cited by Lombardini (1964), who did mention the work of Atyeo (1960) in describing *C. veneta* from Jesolo (Italy). There is a strong need to re-examine specimens attributed by Grandjean (1938) to the undescribed species with leg trichobothria absent) so that the status of the species currently included in the species group *veneta* can be validated.

*Cyta akhyanii* was described based on all developmental stages, except the larva and protonymph, and was collected from sandy soil in Iran (Paktinat-Saeij and Kazemi 2025). The species was reported to be unique from all other known *Cyta* species due to the absence of trichobothria on all legs. Wallace and Mahon (1972) considered dividing the then-known *Cyta* species into three groups based on the number of leg trichobothria. These authors suspected that an unidentified *Cyta* species in their collection belonged to the group with leg trichobothria absent. Earlier, *C. grandjeani* was reported with the absence of all leg trichobothria. Interestingly, this species was not mentioned by either Wallace and Mahon (1972) or Paktinat-Saeij and Kazemi (2025). The latter authors (Paktinat-Saeij and Kazemi 2025) only differentiated *C. akhyanii* from *C. latirostris* by the absence of leg trichobothria and the number of setae on palp-basifemur-genu. These diagnostic characteristics bring the *C. grandjeani* close to *C. akhyanii* in the currently proposed *veneta* species group; in both, leg trichobothria are absent and palp-basifemur and genu have four and three setae, respectively. In the present work, *C. akhyanii* is only suspected to be a junior synonym of *C. grandjeani*. To date, *C. grandjeani* remains poorly described and without diagnostic morphological features figured. As has already been suggested, a detailed description and illustration through re-examination of *C. grandjeani* type material and the unidentified specimens of Wallace and Mahon (1972) is needed.

### Species excluded from species groups and the identification key

*Cyta flava* was described only briefly and without illustrations (Mihelčič 1958). The morphological characters provided were restricted to the body length and width (600–700 µm and 420–500 µm, respectively), colour of body, legs, and eyes, length and width of chelicera (180 µm and 90 µm, respectively), and length of the palp (287 µm) and of palp segments 2^nd^ and 5^th^ (90 µm and 100 µm, respectively). The species was distinguished from *C. latirostris* by having a thicker mandibular claw than the latter. These characters do not allow for the species to be included in the following diagnostic key, and indeed it was previously excluded in Hernandes et al.’s (2016) key. *Cyta flava* is not assigned to one of the species group in the present study. Although the difference between *C. flava* and *C. latirostris* appears to be simply variation, the type specimen examination is recommended for further consideration.

*Cyta cytoides* was originally described in the subgenus *Cytobdella*, then a subgenus of *Bdella* (Mihelčič 1958) but later synonymized with *Cyta* (Atyeo 1963). This species was transferred to *Cyta* on the basis of the massive, stout chelicerae (Hernandes et al. 2016). The original description of *C. cytoides* was poor, and only the propodosoma and chelicera were illustrated. Additionally, later taxonomic works did not provide diagnostic characters for the species, which made it difficult to include it in the current diagnostic key or any species group proposed in the present study.

### Key to known *Cyta* species

Excluding *C. cytoides*, *C. flava*, and *C. grandjeani*.

**Table:** 

1	Leg trichobothria absent	**2 (species group *veneta* )**
–	Leg trichobothria present	**3**
2	Palp basifemora I–III 8–8(9)–6; fixed cheliceral digit with 3 teeth	***C. akhyanii* Paktinat-Saeij & Kazemi, 2025**
–	Palp basifemora I–III 6–6–6; fixed cheliceral digit without tooth	***C. veneta* (Lombardini, 1960)**
3	Leg trichobothrium present only on tibia IV	**4 (*latirostris* species group)**
–	Leg trichobothrium present on tibiae I, IV, and tarsus III	**10 (*coerulipes* species group)**
4	Eyes absent; posterior trichobothria (*pt*) spatulate	***C. magdalenae* Den Heyer, 1981**
–	Eyes present; posterior trichobothria (*pt*) setiform	**5**
5	Coxa IV with 3 setae	**6**
–	Coxa IV with 1–2 setae	**9**
6	Trochanter I with a seta	***C. kurdistanicus* Eghbalian, Khanjani & Ueckermann, 2014**
–	Trochanter I with 2 setae	**7**
7	Moveable cheliceral digit edentate; fixed digit with a tooth	**8**
–	Moveable cheliceral digit with a tooth; fixed digit with 2 teeth	***C. kreiteri* Barbar & Ueckermann, 2017**
8	Dorsal integument entirely with broken striae, palp basifemur with 5 setae	***C. edafosa* sp. nov**.
–	Dorsal integument with broken striae only between dorsocentral setae; palp basifemur with 6 setae	***C. grandjeani* Wu & Guo in Wu et al. 2021**
9	Coxae I–II with 5 setae each; genu IV without attenuate sensory seta	***C. latirostris* (Hermann, 1804)**
–	Coxae I–II with 3/4–2/3 setae; genu IV with attenuate sensory seta	***C. kauaiensis* Swift & Goff, 1987**
10	Coxae and subcapitulum with reticulations	***C. reticulata* Soliman & Zaher, 1975**
–	Coxae and subcapitulum without reticulations	**11**
11	Median proterosomal setae (*mps*) short, not reaching base of lateral proterosomal setae (*lps*)	**12**
–	Median proterosomal setae (*mps*) long, reaching or crossing base of lateral proterosomal setae (*lps*)	**14**
12	Distance between *at*–*at* approximately equal to *pt*–*pt*	***C. brevipalpa* Ewing, 1909**
–	Distance between *at*–*at* about 1/3 to *pt*–*pt*	**13**
13	Palp basifemur with 5 setae; palp genu with 3 setae	***C. arabica* Mirza & Alatawi, 2026**
–	Palp basifemur and genu with 4 setae each	***C. americana* (Banks, 1902)**
14	Setae *at*, *lps*, and *mps* forming the vertices of a triangle, not aligned in a diagonal line	**15**
–	Prodorsal setae *at*, *lps*, and *mps* aligned in almost a transverse line	**17**
15	Distal cheliceral seta minute	***C. ignea* (Tseng, 1978)**
–	Distal cheliceral seta normal and/or longer than cheliceral digit	**16**
16	Trochanter I with 2 setae; genu I with 2 solenidia	***C. coerulipes* (Dugès, 1834) (including subspecies *C. c. quadrisetosus* Den Heyer, 1981)**
–	Trochanter I with a seta, genu I with 3 solenidia	***C. leiliae* Eghbalian, Khanjani & Ueckermann, 2014**
17	Dorsal setae serrate	***C. murrayi* Den Heyer, 1981**
–	Dorsal setae smooth	**18**
18	Palp basifemur with 4 setae	**19**
–	Palp basifemur with 7 setae	***C. longiseta* Wallace & Mahon, 1972**
19	Dorsal striations strong and continuous	***C. troglodyta* Hernandes in Hernandes et al. 2011**
–	Dorsal striations faint and broken	***C. spuria* Atyeo, 1960**

## Supplementary Material

XML Treatment for Cyta

XML Treatment for Cyta edafosa

XML Treatment for Bdella

XML Treatment for Bdella tropica

XML Treatment for Hexabdella

XML Treatment for Hexabdella maraugia

XML Treatment for Odontoscirus

XML Treatment for Odontoscirus longirostris

XML Treatment for Spinibdella

XML Treatment for Spinibdella tabarii

XML Treatment for Spinibdella tadjikistanica

XML Treatment for Spinibdella thori

XML Treatment for Spinibdella subrufa

XML Treatment for Tetrabdella

XML Treatment for Tetrabdella neotropica

## Additional information

### Artificial Intelligence (AI) use

The authors accept full responsibility for the content of the manuscript, including the disclosure of any use of AI.

No AI tools were used in the preparation of this manuscript.

### Author ORCIDs

J.H. Mirza https://orcid.org/0000-0002-1726-4331

F.J. Alatawi https://orcid.org/0000-0002-6824-2650

### Data availability

All of the data that support the findings of this study are available in the main text.
